# Tissue effects of a newly developed diode pumped pulsed Thulium:YAG laser compared to continuous wave Thulium:YAG and pulsed Holmium:YAG laser

**DOI:** 10.1007/s00345-021-03634-4

**Published:** 2021-03-16

**Authors:** Stephan Huusmann, Marcel Lafos, Ingo Meyenburg, Rolf Muschter, Heinrich-Otto Teichmann, Thomas Herrmann

**Affiliations:** 1 Department of Urology and Urologic Oncology, Hannover Medical School, Carl-Neuberg-Str. 1, 30625 Hannover, Germany; 2 Hannover Medical School, Institute for Pathology, Hannover, Germany; 3 LISA Laser Products GmbH, Katlenburg-Lindau, Germany; 4 Alta Clinic, Bielefeld, Germany; 5 Clinic for Urology, Spital Thurgau, Thurgau, Switzerland

**Keywords:** Pulsed Thulium:YAG laser, Continuous wave Thulium:YAG laser (CW), Holmium:YAG laser, Tissue interaction

## Abstract

**Purpose:**

The objective of this study is to evaluate the laser-tissue effects of laser radiation emitted by a newly developed high frequency pulsed Tm:YAG laser in comparison to the continuous wave Tm:YAG laser and the pulsed Ho:YAG laser.

**Methods:**

Ex-vivo experiments were performed on freshly slaughtered porcine kidneys in a physiological saline solution. Experiments were performed using two different laser devices in different settings: A Tm:YAG laser was operated in a pulsed mode up to 300 Hz and in a continuous wave (CW) mode. Results were compared with a 100 W standard pulsed Ho:YAG laser system. Comparative tissue experiments were performed at 5 W, 40 W and 80 W. The incision depth and the laser damage zone were measured under a microscope using a calibrated ocular scale.

**Results:**

Increased laser power resulted in increased incision depth and increased laser damage zone for all investigated lasers in this set-up. The Ho:YAG created the largest combined tissue effect at the 5 W power setting and seems to be the least controllable laser at low power for soft tissue incisions. The CW Tm:YAG did not incise at all at 5 W, but created the largest laser damage zone. For the new pulsed Tm:YAG laser the tissue effect grew evenly with increasing power.

**Conclusion:**

Among the investigated laser systems in this setting the pulsed Tm:YAG laser shows the most controllable behavior, insofar as both the incision depth and the laser damage zone increase evenly with increasing laser power.

**Supplementary Information:**

The online version contains supplementary material available at 10.1007/s00345-021-03634-4.

## Introduction

In the last three decades, several different laser technologies have been introduced and have found their applications in surgical procedures [1–4]. For urologic surgery the Holmium:YAG laser (Ho:YAG) and the Thulium:YAG laser (Tm:YAG) have gained the most recognition. Both lasers emit in the 2 µm wavelength range. Because of the similarity in wavelength, the assumption prevails that the laser-tissue effect of the mentioned devices is similar as well [5]. However, pulsed Ho:YAG and continuous wave (CW) Tm:YAG create different tissue effects due to different emission modes. While the pulsed Ho:YAG laser tears and pushes tissue mechanically by a rapidly growing and collapsing steam bubble, the CW Tm:YAG laser cuts tissue by continuous vaporization of aqueous tissue constituents. Despite the lack of data previous studies show that the CW Tm:YAG laser has a stronger vaporization and a better hemostatic effect [6–8].

A promising further development for the Tm:YAG laser is the addition of a pulsed emission mode to combine the strong vaporization effect of the Tm:YAG laser with the properties of the Ho:YAG laser. A recently available technical modification allows the Tm:YAG laser to be operated in either continuous wave mode or pulsed emission mode.

This study aims to compare the tissue effects of the newly developed pulsed Tm:YAG laser with the established effects of CW Tm:YAG laser and the pulsed Ho:YAG laser.

## Methods

The newly developed laser is a diode pumped Tm:YAG laser, which emits laser radiation at a wavelength of 2013 nm. The technical realization allows multiple operating modes. For our experiments, a *RevoLix HTL* prototype (LISA Laser Products GmbH) was operated in CW and in pulsed mode up to 300 Hz with a pulse peak power exceeding the power of the CW operation. The adjustable parameters on this device are the output power (W) in CW mode, and in the pulsed mode pulse peak power (W), pulse duration (µs) and pulse frequency (Hz). In each operational mode, the average output power from the laser fiber was measured by a calibrated external power meter (Ophir Energy Sensor Head).

As a reference, a 100 W Ho:YAG laser system (Sphinx 100 W, LISA Laser Products GmbH) was used. Ho:YAG lasers are excited by flash lamps. Consequently, they operate in a pulsed mode only. The output of the Ho:YAG laser is set separately for pulse energy in Joule (J) and pulse repetition rate in Hertz (Hz) leading to the output power in Watt (W). The pulse duration was set to 250 µs for all experiments with the Ho:YAG laser.

For all experiments, a freshly cleaved laser fiber with an optical core diameter of 550 µm (*RigiFib*, LISA Laser Products GmbH) was used.

We selected different levels of laser power for the experiments: 5 W, 40 W and 80 W.

Kidneys were harvested from freshly slaughtered pigs and stored at 2–5 °C until the experiments were performed. For the experiments, they were cut into bars which were fixed (superglue) on a specimen holder and placed in a bath of room tempered physiological saline solution. The laser fiber was fixed in an applicator (*SurgiLas L50*, LISA Laser Products GmbH) at an angle of 45°. The applicator was attached to a computer-controlled motorized xyz-stage. The laser fiber was placed with the tip in contact with the surface of the specimen (Supplementary Fig. 1).

The motorized stage was programmed for a continuous linear motion at 2 mm/s across the specimen.

All results shown are the arithmetic mean of 3 identical experiments.

### Histological evaluation

For histological evaluation, the specimens were fixed in 4% formalin solution and finally embedded in paraffin. Histological sections with a thickness of 2–3 µm were prepared and stained with hematoxylin and eosin stain. The incision depth and the width of the laser damage zone were measured under a microscope using a calibrated ocular scale.

### OC-zone, NT-zone, E-zone (Fig. 1)

**Fig. 1:**
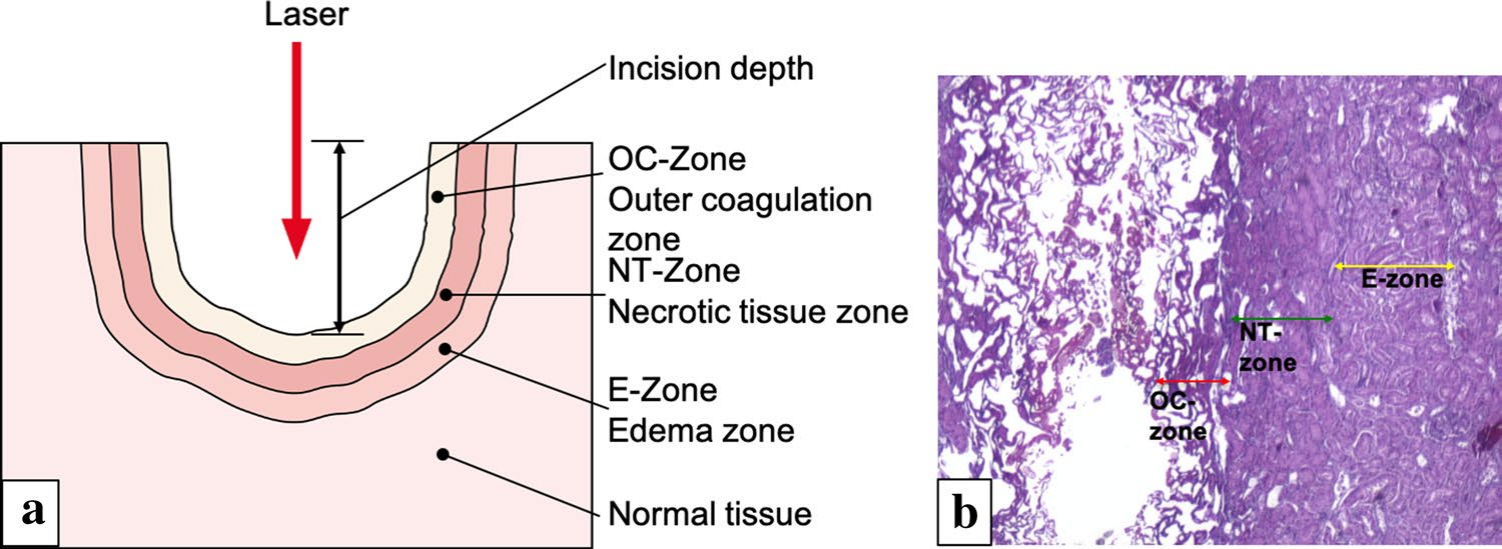
** a** Nomenclature of laser affected tissue layers after laser irradiation. **b** Histologic section of a laser cut in the porcine kidney with pulsed Tm:YAG at 80 W with marked damage zones

The outer coagulation zone (OC-zone) is characterized by a carbonized seam and a tissue layer with vacuolization underneath. Here the cell and tissue structure largely cannot be recognized. In hematoxylin and eosin staining, the OC-zone appears dark purple (Fig. 1). The necrotic tissue layer (NT-zone) is characterized at high magnification by pycnotic nuclei of the cells. The underlying edema zone (E-zone) results from an exposure to heat generated from absorbed laser energy. In vivo, the E-zone has the potential to recover thus it is not considered to be part of the laser damage zone (Fig. 1).

The laser damage zone is defined as the outer coagulation zone plus the necrotic zone [10] (Fig. 1). The intentionally created incision depth and the laser damage zone together are therefore the clinically significant tissue damage.

This study evaluates the incision depth and the laser damage zone due to the absorbed laser power. The results indicate a dependence between the clinically significant tissue damage and the investigated laser system used under the above-mentioned set-up.

## Results

**Fig. 2:**
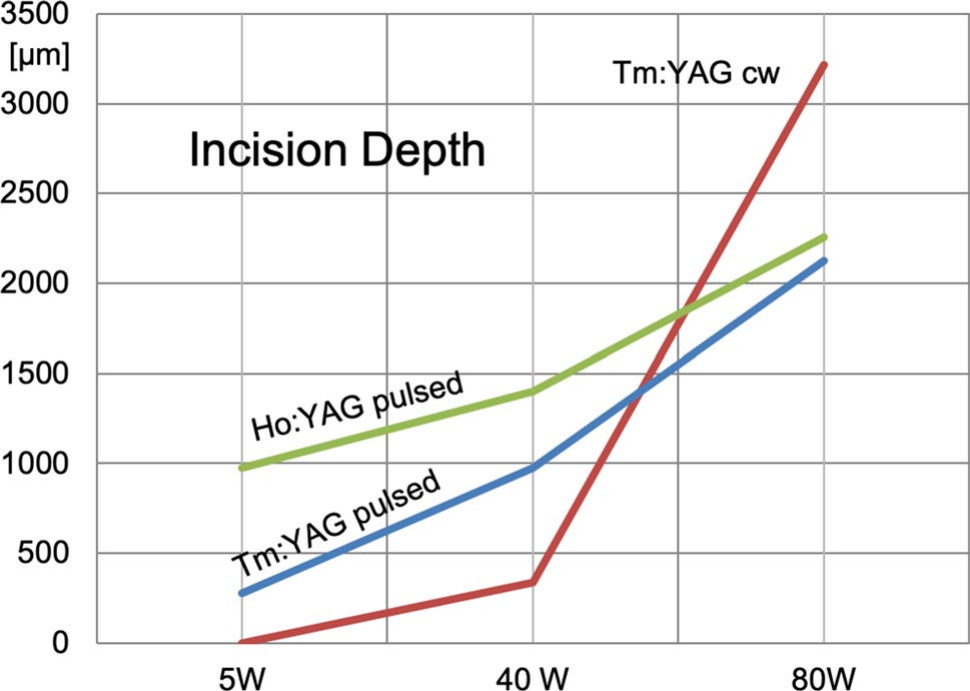
Incision depth of CW Tm:YAG, pulsed Tm:YAG and pulsed Ho:YAG at different laser energy levels

**Fig. 3:**
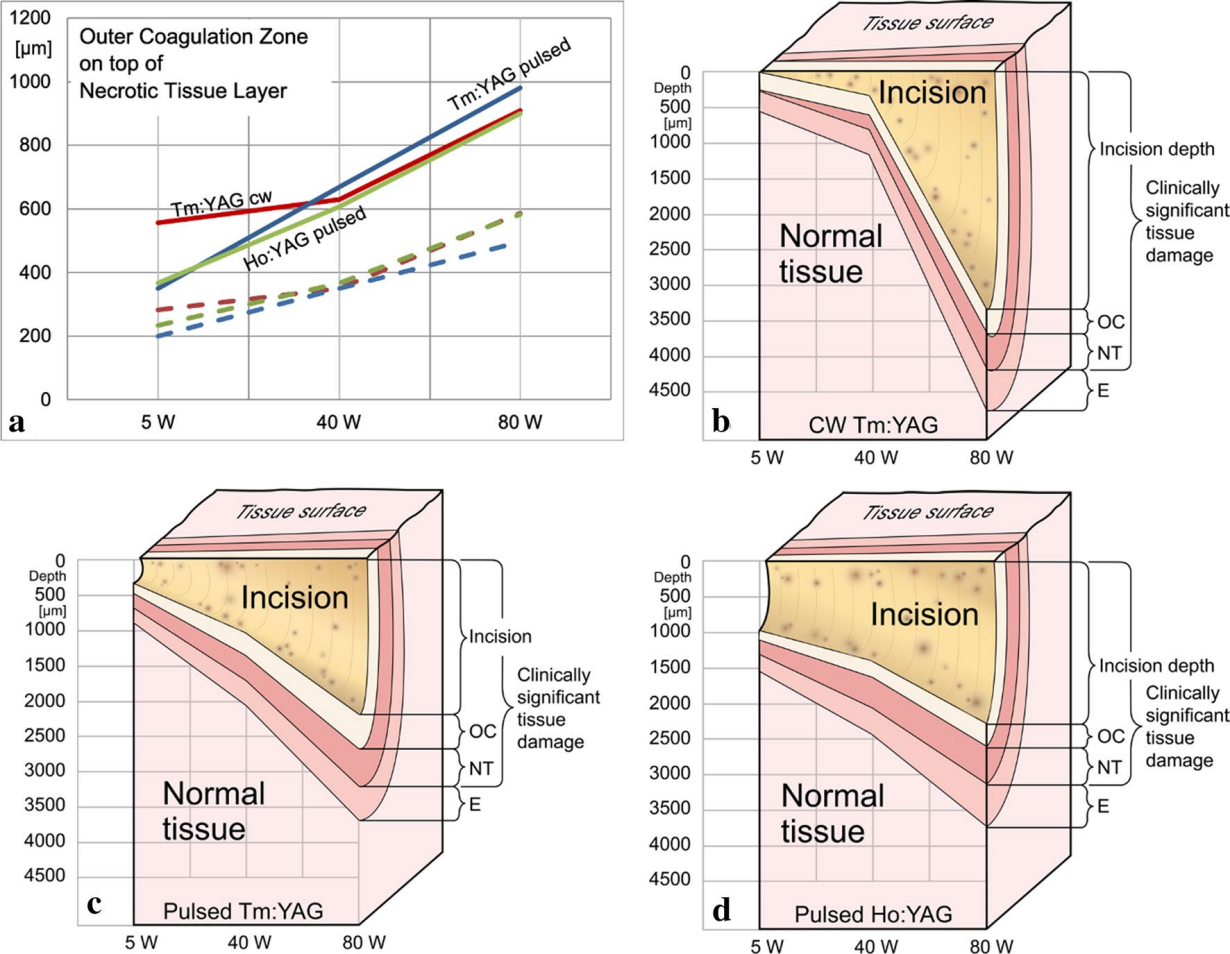
Laser damage zones for the different laser devices (**a**). Graphically added incision depth for Tm:YAG CW (**b**), pulsed Tm:YAG (**c**), pulsed Ho:YAG (**d**)

The results are summarized in Table 1 and illustrated in Figs. 2 and 3.

**Table 1:**
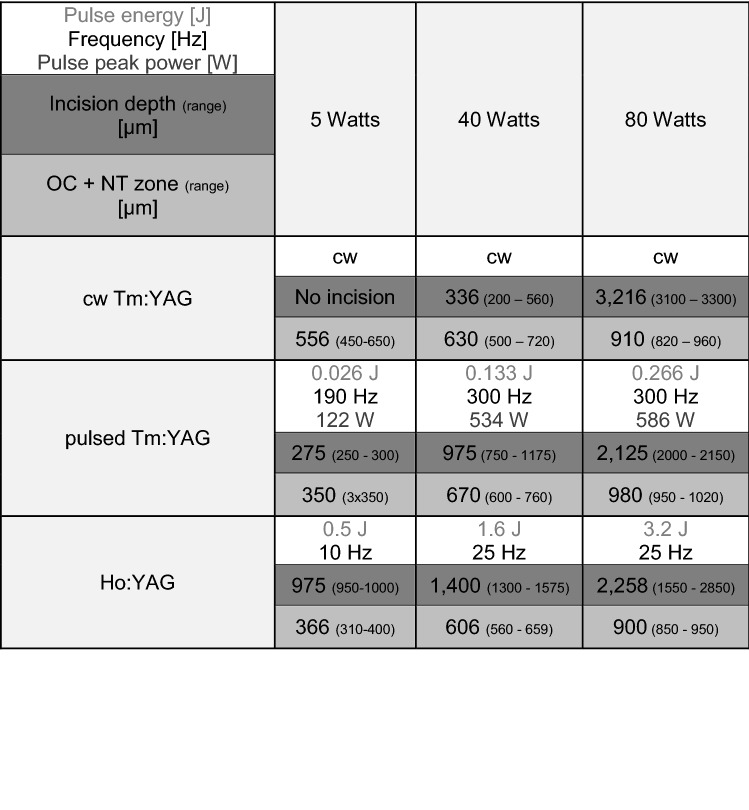
Clinically significant tissue damage: incision depth and laser damage zone OC + NT (given values correspond to the mean of 3 histological sections)

At 5 W the incision depth was 275 µm (250–300) for the pulsed Tm:YAG, 0 µm (no tissue cut visible) for the CW Tm:YAG, and 975 µm (950–1000) for the pulsed Ho:YAG. The laser damage zone (OC + NT) at this setting was measured to be 350 µm for the pulsed Tm:YAG, 556 µm (450–650) for CW Tm:YAG and 366 µm (310–400) for Ho:YAG.

At 40 W, the incision depth was 975 µm (750–1175) for the pulsed Tm:YAG, 336 µm (200–560) for the CW Tm:YAG and 1400 µm (1300–1575) for the Ho:YAG. The laser damage zone in this setting showed a depth of 670 µm (600–760) for the pulsed Tm:YAG, 630 µm (500–720) for the CW Tm:YAG and 606 µm (560–659) for the Ho:YAG.

At 80 W, the incision depth was 2125 µm (2000–2150) for the pulsed Tm:YAG, 3216 µm (3100–3300) for the CW Tm:YAG, and 2258 µm (1550–2850) for the Ho:YAG. The laser damage zone in the high-power setting showed a depth of 980 µm (950–1020) for the pulsed Tm:YAG, 910 µm (820–960) for the CW Tm:YAG and 900 µm (850–950) for the Ho:YAG (Fig. 3).

Moreover, there are macroscopically observable differences in the incisions between the different lasers. As seen in the supplementary, the Ho:YAG laser produces a coarse cut in the tissue without carbonization (SuppFig. 2C) whereas the CW Tm:YAG laser creates a smooth cut with continuous caramel to dark carbonization (Supp Fig. 2A). Macroscopically, the pulsed Tm:YAG laser creates a smooth cut with light caramel coloration and little charring (Supp Fig. 2B).

## Discussion

The Ho:YAG laser plays a decisive role in laser enucleation of prostate (HoLEP) [9, 10]. In this application, the CW Tm:YAG laser has become a serious alternative to the Ho:YAG laser [6, 11]. Both systems emit laser energy at a wavelength of around 2000 nm (Ho:YAG at 2123 nm and Tm:YAG at 2013 nm), which is close to the 2 µm absorption maximum of liquid water. Consequently, the optical absorption is very strong in water and aqueous solutions, leading to a shallow optical penetration of laser radiation in soft tissue [12, 13]. This concentrates the laser effect to the surface of the irradiated tissue and prevents deep coagulation, thus increasing the safety of the laser procedure [14]. Due to the minor difference in optical penetration and the potential advantage of the continuous wave mode, the Tm:YAG laser may have advantages in terms of coagulation, hemostasis and vaporization of tissue [6].

The technical modification allows the Tm:YAG laser to be operated not only in a CW mode but also in a pulsed mode which may combine the advantageous properties of both laser systems into one device while leaving behind previously existing undesirable or adverse laser properties.

The objective was to compare incision depth and laser damage zones in vitro between CW Tm:YAG, pulsed Tm:YAG and Ho:YAG.

The outer coagulation zone (OC-zone) and the necrotic zone (NT-zone) together represent irreversible thermal tissue damage [15]. This damage zone is related but not identical to the absorption length at the laser wavelength in water. At the Tm:YAG laser wavelength of 2013 nm, the absorption length in water is 165 µm and approximately 426 µm at the Ho:YAG wavelength of 2123 nm [13]. For surgical laser applications, absorption of light is the most important element of light-tissue-interaction. Previous comparative studies have shown that the laser damage zone for Ho:YAG (447 µm at 5 W, 677 µm at 80 W [7]) and cw Tm:YAG (550 µm at 5 W, 653 µm at 120 W [7, 16], 1090 µm at 70 and 120 W [15]) depending on laser power up to about 80 W and 120 W for Ho:YAG and Tm:YAG, respectively, is in an acceptable range of approximately one millimeter [7, 15]. Our current experiments confirmed these results for the Ho:YAG and the Tm:YAG laser (CW and pulsed). We did not detect significant differences in the newly developed Tm:YAG laser concerning the laser damage zone in either operating mode (CW or pulsed) except for the CW Tm:YAG at 5 W where we observed no incision and a deep laser damage zone. For all investigated laser sources, the width of the laser damage zone increased evenly with increasing power (see Fig. 3).

When comparing the observed power dependency of the laser damage zone with findings in [15] where a power independent damage zone is described the difference in the experimental set-up needs to be considered: The authors of [15] applied the Tm:YAG laser radiation to perfused kidneys. The perfusion supports the heat flow from the laser cut downwards into the unaffected tissue, thus providing a cooling effect which reduces the heat damage.

Another difference is in the applied power level: This study investigated incision depth and laser damage zone at a lower power range (5–80 W) whereas in [15] the applied power range was at a higher level (70 and 120 W).

Consequently, we anticipate comparable properties of the new laser regarding coagulation, hemostasis and vaporization and tissue damage in vivo.

Regarding the incision depth (see Fig. 2) the new pulsed Tm:YAG laser shows comparable results to previously examined laser systems [7, 15]. In the pulsed mode, the incision depth of the Tm:YAG laser increases evenly with the laser power from 275 µm at 5 W up to 2125 µm at 80 W whereas the Ho:YAG laser has a similarly even increase but starting from a higher value of 975 µm incision depth already at 5 W.

For the CW Tm:YAG laser the non-linear increase of the incision depth (3216 µm at 80 W) with increasing power stands out.

Visual comparisons of the histological sections suggest that the pulsed mode of the Tm:YAG laser can produce effects similar to those of a Ho:YAG laser (see Supplementary Fig. 2). The macroscopic appearance of the sections and the microscopic effects examined here suggest that the pulsed Tm:YAG laser may combine the hemostasis, coagulation and vaporization properties of the Tm:YAG laser with the mechanically pushing and tearing properties of the Ho:YAG laser. In further experiments, the goal should be to find settings for the pulsed Tm:YAG that feature a Ho:LEP-like push and tear effect on the cleavage between adenoma and capsule without the excessive mechanical tearing and vibration which is typical for the Ho:YAG laser. Additionally, despite research showing that laser tissue damage can be estimated well using cadaveric porcine kidneys, the limitations of cadaveric porcine kidneys for the investigation of laser-tissue damage and incision depth must be accepted. However, laser-tissue damage can be estimated well using porcine kidneys [17, 18]. Undoubtedly, conditions in vivo will deviate due to continuous perfusion, differences of organ-specific tissue properties and in continuous removal of the heat due to irrigation.

## Conclusion

The laser damage zone for all investigated lasers is almost identical except for the cw Tm:YAG laser at low power 5 W (high 556 µm laser damage zone).

In the chosen settings the pulsed Tm:YAG laser creates less carbonization than the CW Tm:YAG laser, less trauma than the Ho:YAG laser and features the most controllable behavior with evenly increasing incision depth and laser damage zone with increasing laser power.

## Supplementary Information

Below is the link to the electronic supplementary material.

Supplementary file1 (TIFF 5931 KB)

Supplementary file2 (TIFF 1567 KB)
