# Supplementary figures and images for: Tissue effects of a newly developed diode pumped pulsed Thulium:YAG laser compared to continuous wave Thulium:YAG and pulsed Holmium:YAG laser
Source: World J Urol. 2021 Mar 16;39(9):3503–8. doi: 10.1007/s00345-021-03634-4 (PMC8510916; doi:10.1007/s00345-021-03634-4)

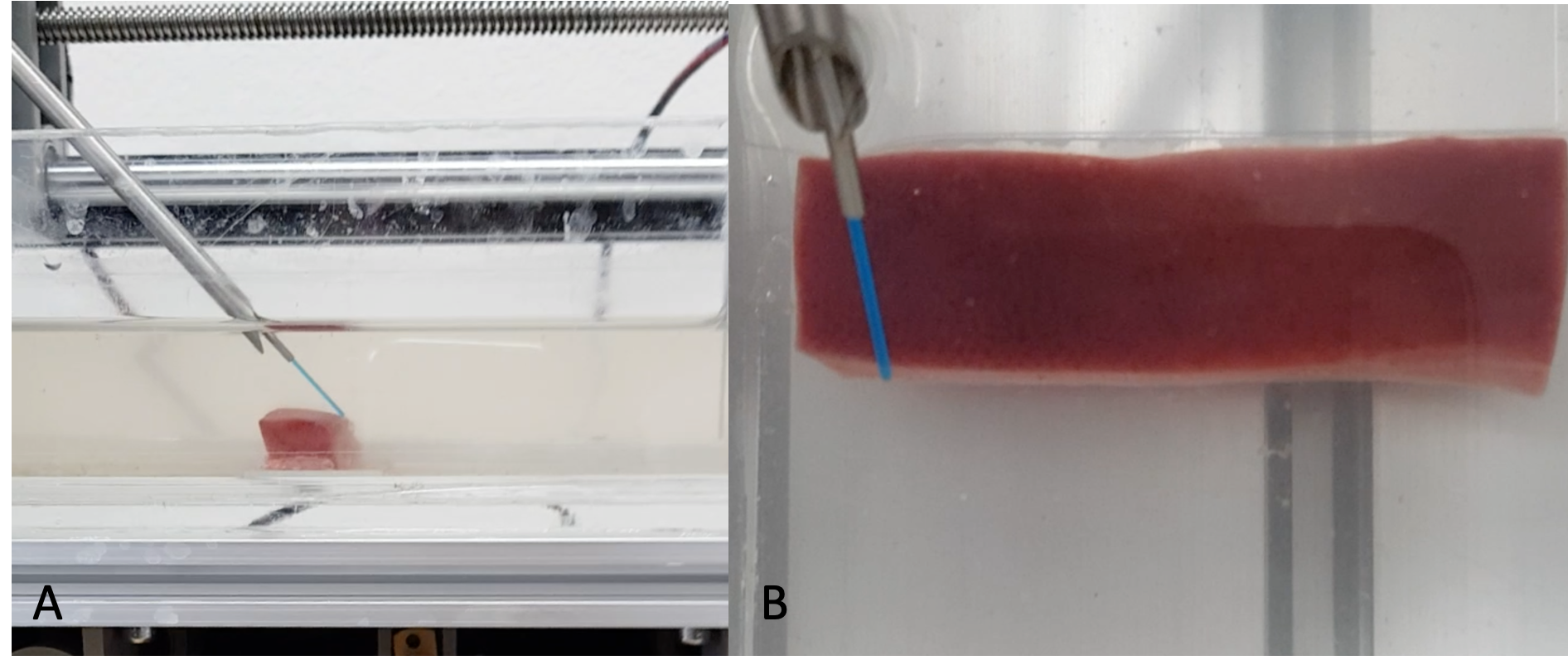

Supplement: Supplementary file 1 — Supplementary file1 (TIFF 5931 KB) [file 345_2021_3634_MOESM1_ESM.tiff]

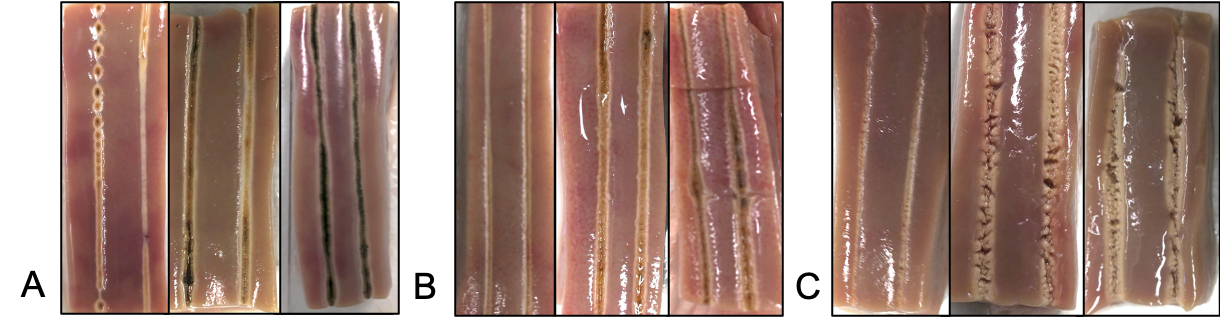

Supplement: Supplementary file 2 — Supplementary file2 (TIFF 1567 KB) [file 345_2021_3634_MOESM2_ESM.tiff]
